# Understanding and Addressing Older Adults’ Loneliness: The Social Relationship Expectations Framework

**DOI:** 10.1177/17456916221127218

**Published:** 2022-11-02

**Authors:** Samia C. Akhter-Khan, Matthew Prina, Gloria Hoi-Yan Wong, Rosie Mayston, Leon Li

**Affiliations:** 1 Department of Health Service and Population Research, Institute of Psychiatry, Psychology and Neuroscience, King’s College London; 2 Department of Social Work and Social Administration, The University of Hong Kong; 3 Department of Global Health and Social Medicine, Institute of Global Health, King’s College London; 4 Department of Psychology and Neuroscience, Duke University

**Keywords:** generativity, healthy aging, personal relationships, interventions, culture

## Abstract

Loneliness is an experience resulting from a perceived discrepancy between expected and actual social relationships. Although this discrepancy is widely considered the “core mechanism” of loneliness, previous research and interventions have not sufficiently addressed what older adults specifically expect from their social relationships. To address this gap and to help situate research on older adults’ loneliness within broader life span developmental theories, we propose a theoretical framework that outlines six key social relationship expectations of older adults based on research from psychology, gerontology, and anthropology: availability of social contacts, receiving care and support, intimacy and understanding, enjoyment and shared interests, generativity and contribution, and being respected and valued. We further argue that a complete understanding of loneliness across the life span requires attention to the powerful impacts of contextual factors (e.g., culture, functional limitations, social network changes) on the expression and fulfillment of older adults’ universal and age-specific relationship expectations. The proposed Social Relationship Expectations Framework may fruitfully inform future loneliness research and interventions for a heterogeneous aging population.

The consensus psychological definition of loneliness is a feeling that results from a perceived discrepancy between desired and achieved levels of social relationships (Peplau & Perlman, 1982). This gap between expected and actual social relationships may be considered the “core mechanism” of loneliness. Importantly, loneliness is not equivalent to social isolation. Whereas social isolation pertains to objective features of one’s situation, such as the number or proximity of social contacts that one has, loneliness is a fundamentally subjective experience (Peplau & Perlman, 1982). Two dimensions of loneliness, a social dimension and an emotional dimension, have often been distinguished (Weiss, 1973). The social dimension refers to expectations about the quantity of relationships (i.e., how many social ties are nearby, available, and can be relied on for support). The emotional dimension refers to expectations about the quality of relationships, such as the degree of intimacy, understanding, and interests that one shares with others (Peplau & Perlman, 1982). Both dimensions have been included in prominent scales of loneliness, such as the De Jong Gierveld (DJG) Loneliness Scale (De Jong Gierveld & Van Tilburg, 2006).

Loneliness is a natural feeling that occurs occasionally in most people’s lives, and it serves evolutionarily adaptive functions, such as motivating people to maintain important relationships (Cacioppo et al., 2006, 2014). However, when loneliness becomes a persistent or chronic state, it may have adverse effects on health and well-being (Cacioppo et al., 2014). For example, persistent loneliness has been shown to be an independent risk factor for developing depression, cognitive impairment, dementia, Alzheimer’s disease, and all-cause mortality (Akhter-Khan et al., 2021; Martín-María et al., 2021; Shiovitz-Ezra & Ayalon, 2010; Zhong et al., 2016). Efforts and interventions to prevent and address chronic loneliness and its adverse effects would benefit substantially from a solid theoretical foundation that comprehensively describes the causes, contextual factors, and avenues for addressing loneliness (Akhter-Khan & Au, 2020; Gardiner et al., 2018; O’Rourke et al., 2018). However, to date, there has been no comprehensive framework specifying the contents that constitute the core mechanism of loneliness (i.e., what people specifically expect from their social relationships) or the cultural and life span developmental factors that affect the functioning of this core mechanism.

Taking a life span perspective on loneliness is important because the prevalence, stability, risk factors, and consequences of loneliness differ between age groups (Beam & Kim, 2020; Dykstra, 2009; Luhmann & Hawkley, 2016; Qualter et al., 2015). Research has shown that the prevalence of loneliness follows a U-shaped distribution, with the highest rates in late adolescence and young adulthood, lower rates during midlife, and another peak among the oldest adults (80+ years; Beam & Kim, 2020; Dykstra, 2009; Qualter et al., 2015). Whereas between 3% and 22% of people experience persistent loneliness in early childhood or young adulthood, longitudinal studies report that 15% to 25% of older adults experience persistent levels of social or emotional isolation (Qualter et al., 2015). A recent meta-analysis indicated greater interindividual differences in loneliness over a 5-year trajectory among the oldest adults compared with other age groups (Mund et al., 2020). Greater heterogeneity in the development of loneliness in older adults may be due to more fragile environmental niches that may change with impaired health status or the loss of important relationships (Mund et al., 2020). A study with representative data from Germany also found that relationship status was an age-specific predictor for loneliness, showing more predictive value for middle-aged and older adults as opposed to younger adults (Luhmann & Hawkley, 2016).

Long-term adverse health outcomes related to loneliness, such as cardiovascular disease and dementia, are likely to occur towards the end of the life span (Ong et al., 2016; Valtorta et al., 2016). Moreover, given that the future number of individuals who feel lonely may increase with a growing “oldest old” population (Hawkley et al., 2019), finding solutions to (chronic) loneliness should be viewed as a priority during the United Nations (UN) Decade of Healthy Aging (2021–2030; World Health Organization, 2020), a UN initiative to improve older people’s well-being amid population aging. To date, interventions to reduce loneliness have not been as effective as interventions for other social and behavioral outcomes (Masi et al., 2011). For example, a meta-analysis by Masi et al. (2011) reported an effect size of only −0.198 for randomized studies (equivalent to a 1.59-point reduction on the 20-item UCLA Loneliness Scale), which does not represent a clinically significant reduction in loneliness to the levels found in “healthy, community-living individuals” (Masi et al., 2011, p. 257). More recent meta-analyses have reported no effect in reducing loneliness (Shah et al., 2021) or small to medium effect sizes for psychotherapy (Hickin et al., 2021). Addressing the problem of chronic loneliness may require a deeper theoretical understanding of how age-related contextual factors relate to loneliness. Although there have been many important life span developmental theories about aging and age-related contextual factors, they have not been linked to older adults’ loneliness in a comprehensive framework.

To address the existing gaps in the literature, the current article aims to (a) describe how the core mechanism of loneliness is situated within age-related contextual factors that have been described by prominent life span developmental theories (Fig. 1) and (b) specify the universal and age-specific types of relationship expectations that older adults have (Fig. 2). The Social Relationship Expectations (SRE) Framework that is introduced may help to illuminate how older adults’ universal and age-specific relationship expectations manifest across different cultural contexts, as well as offer implications for future research and intervention targets.

**Fig. 1.**
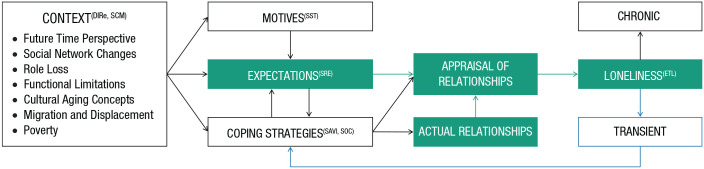
The core loneliness mechanism situated in life span developmental theories. According to the “core mechanism” of loneliness (highlighted in green), loneliness results from a discrepancy between expected and actual social relationships, as assessed by one’s appraisal of one’s relationships. Prominent life span developmental theories have described how context, motives, expectations, and coping strategies, as well as their interrelations, change with age. Loneliness may be transient when coping strategies can be successfully executed to modify one’s expectations, appraisals, or actual relationships (blue pathway). In certain situations or contexts, however, such coping strategies cannot be applied, resulting in chronic loneliness. DIRe: Differential Investment of Resources Model. SCM: Social Convoy Model. SST: Socioemotional Selectivity Theory. SRE: Social Relationship Expectations Framework. SAVI: Strength and Vulnerability Integration Model. SOC: Selective Optimization with Compensation Theory. ETL: Evolutionary Theory of Loneliness.

**Fig. 2.**
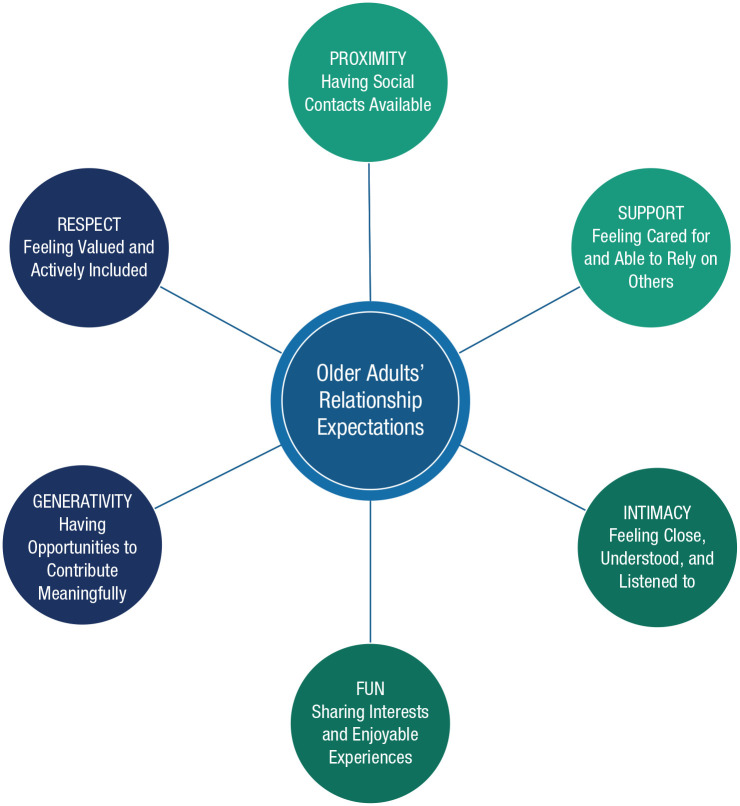
Older adults’ social relationship expectations (SRE). Older adults have four relationship expectations that are universal (proximity, support, intimacy, and fun) and two that are age-specific or have age-specific manifestations (generativity and respect).

## Contextualizing the Core Mechanism of Loneliness in Life Span Developmental Theories

Aging has been aptly described as “an iterative, socially embedded process that requires adaptation to specific sociocultural contexts” (Perkinson & Solimeo, 2014, p. 102). However, little research has explored how sociocultural context impacts the manifestation of loneliness, possibly because of a general bias within psychological research to focus on higher-income contexts (Henrich et al., 2010). Here, we aim to address this gap by contextualizing the core mechanism of loneliness within situated aging processes. Figure 1 illustrates how people’s social relationship expectations are situated in age-related and contextual factors, motives, and coping strategies that have been described in depth by previous life span developmental theories (e.g., Antonucci et al., 2014; Baltes, 1997; Carstensen, 1993; Charles, 2010; Huxhold et al., 2022).

### Situating loneliness in age-related contexts

Loneliness differs between younger and older adults, yet there are also many other factors aside from chronological age that affect the core mechanism of loneliness. People’s expectations for their social relationships, as well as their success at realizing their expectations, are contingent on a variety of external factors, such as living arrangements (e.g., long-term care institutions vs. aging in place), resources (e.g., abundance of activities vs. scarcity), and structural changes that may cause migration or displacement (e.g., war or climate change). Furthermore, there are certain changes to contextual factors that are likely to accompany old age, such as a decline in physical health, losses to social networks, and shifts in cultural expectations resulting from retirement, filial piety, and ageism in people’s environments. In the following section, we review relevant developmental theories and give examples of how contextual factors can affect people’s motives, coping strategies, expectations, and efforts to fulfill expectations.

One influential life span developmental theory describing contextual influences on social relationships is the Social Convoy Model (SCM; Antonucci & Akiyama, 1987, 1991; Antonucci et al., 2014). The SCM describes how features of social networks, such as the structure, function, and quality of social relationships, change with personal and situational characteristics (Antonucci et al., 2014). Social relations are affected by factors such as “relationship type (i.e., spouse, friend), gender, age, contact frequency, and geographical proximity” (Antonucci et al., 2014, p. 84). Circumstances that increasingly arise with age, such as functional limitations or role loss resulting from retirement, change older adults’ expectations for receiving social support and affect the longer-term consequences of (perceived and actual) social support for health and well-being (Antonucci et al., 2014). In addition, older adults’ provision of support to others has been associated with increased well-being and lower loneliness (Antonucci et al., 2014; De Jong Gierveld & Dykstra, 2008; De Jong Gierveld et al., 2012), implying that older adults expect certain social opportunity structures that allow providing as well as receiving support depending on the individual’s circumstances. A recent theoretical model, the Differential Investment of Resources (DIRe) Model, describes how social opportunity structures change with age and how people’s strategic investments of time and energy into different social relationships change in response (Huxhold et al., 2022). Older people generally concentrate their time and energy into developing closer relationships, but there is also interindividual variability, as individuals’ investment decisions may be driven by various goals (or expectations) for specific relationships depending on their living situation, social structures, and norms (for a detailed review, see Huxhold et al., 2022).

Of course, cultural expectations and norms are strong contextual factors affecting all aspects of older adults’ social relationships (e.g., motives, expectations, and coping strategies), including expectations about aging (e.g., ageism) and preferred living arrangements. For example, older people in Northern European countries tend to expect independence and are not necessarily lonely when living alone, whereas older people in Southern European countries, where familism and communality are more highly valued, have greater expectations of co-residence with children (Dykstra, 2009). These differences in expectations may potentially explain why the prevalence of loneliness has not increased over time despite increasing individualism (especially in higher-income countries; Drewelies et al., 2019; Hawkley et al., 2019). If older people expect to be alone, they do not feel as disappointed by sparser relationships (Dykstra, 2009).

The core mechanism of loneliness is also shaped by cultural norms about social opportunity structures. For instance, a recent study conducted in four European countries showed that cultural norms that encouraged the creation of new relationships were associated with lower loneliness (Heu et al., 2021). Moreover, when people are displaced and migrate (e.g., because of natural disasters or political conflicts), they may be confronted with different cultural relationship expectations in the new setting, which may result in unfulfilled expectations, stress, and loneliness (Albert, 2021; Wilmoth & Chen, 2003). Altogether, these examples illustrate how culture may shape expectations regarding living arrangements and relationships. Throughout this article, we give further examples of how culture and contexts shape the contents of relationship expectations. First, however, we elaborate on how life span developmental theories describe age-related changes in motives and coping strategies following a perceived limited future time perspective and a loss of functional abilities.

### Motives

The aging process usually goes hand in hand with losses pertaining to functional and cognitive abilities, roles and status, and one’s quantity of relationships. According to the Socioemotional Selectivity Theory (SST), older adults compensate for these losses in part by shifting their motives (Carstensen, 1993, 2006; Carstensen et al., 1999). A limited future time perspective compels people to prioritize certain relationships on the basis of an increased pursuit for meaningful interactions that fulfill a sense of purpose (Carstensen, 1993). This motivational shift has been empirically confirmed in several studies that have shown that people with a constrained time perspective (including younger adults with chronic illnesses) engage more in emotional goals as opposed to knowledge-related goals (Carstensen et al., 1999; Carstensen & Mikels, 2005). In turn, a shift in motives towards more meaningful relationships may affect what people expect from their relationships. For example, people with limited future time perspectives expect more intimacy in their relationships compared with people with an open-ended time horizon (Chu et al., 2018).

### Coping strategies

Despite experiencing developmental changes such as losses of social relationships, social roles, and functional ability, older adults are often able to remain resilient by employing certain coping strategies. The Selective Optimization with Compensation (SOC) Theory (Baltes, 1997) delineates three broad categories of coping strategies: selection, optimization, and compensation. Selection refers to the strategic selection of goals that can be fulfilled despite one’s constraints. Optimization refers to the allocation of resources to facilitate functioning by applying “cultural knowledge, physical status, goal commitment, practice, and effort” (Baltes, 1997, p. 371). Compensation describes the process of adapting to losses and using substituting processes, such as in response to declines in physical functioning (e.g., hearing loss; Freund & Baltes, 1998). These coping strategies allow older adults to promote their well-being. According to the Strength and Vulnerability Integration (SAVI) Model (Charles, 2010), older adults are generally better than younger adults at regulating their emotions, using attentional strategies that focus on more positive stimuli (i.e., the positivity effect) and engaging in less confrontational behaviors. The SAVI model also proposes that the positivity effect, which has been confirmed in attention and memory tasks (Carstensen & Mikels, 2005), may be due to older adults having a more limited future time perspective, as well as more life experience (Charles, 2010). Relevant to this, experimental research has shown that thinking about a limited future can induce a positivity effect in one’s recall of emotional pictures (Barber et al., 2016).

Although SOC coping strategies have been associated with reduced loneliness in the Berlin Aging Study (Freund & Baltes, 1998), the pathways through which these coping strategies affect loneliness have not been elucidated. Figure 1 shows three possible pathways (as depicted by arrows) between coping strategies and aspects of the core mechanism of loneliness. First, coping strategies could affect the expectations that older adults have (e.g., lowering expectations for peripheral relationships because of a prioritization of one’s more intimate contacts). Second, they could affect older adults’ appraisal of social relationships (e.g., focusing attention and memory on more positive interactions). Third, they could affect how older adults interact with their relationship partners (e.g., using a less confrontational interaction style; Fig. 1). An example may help illustrate these three pathways. During the lockdowns and social distancing measures associated with COVID-19, older people who lived alone or in long-term care facilities were not able to meet friends, families, and neighbors, and they were thus socially isolated for longer periods than usual. However, social isolation is not equivalent to loneliness, and not all older adults who lived alone during the lockdown felt lonely (Luchetti et al., 2020). This may have been due to older adults deploying certain coping strategies to compensate for their social isolation. Namely, older adults may have adjusted their expectations by selecting new goals (e.g., contacting people digitally rather than in person), invested time and energy to optimize their technological skills (e.g., mastering high-touch technology, such as video calls), and valued the few positive interactions that they had with others (the positivity effect).

Importantly, not all older adults have the means to successfully use these coping strategies. For instance, older adults with limited or no access to the Internet or smart technologies, as well as older adults with little technological literacy, may not be able to contact their friends and families during social distancing periods. Indeed, people with lower education levels, poorer health, and limited access to new technologies may not only be the most vulnerable to experiencing loneliness but are also often the very populations that are not represented in most research on loneliness (Dahlberg, 2021). Relevant to this, the SAVI model emphasizes that older people are especially vulnerable to prolonged stressors (Charles, 2010). These considerations imply that chronic loneliness may be a more prominent concern for older adults than for younger adults because older adults may not have the resources (e.g., time, energy, education, digital literacy) and coping strategies to successfully overcome loneliness when affected by vulnerabilities such as impaired health. In the next section, we turn to an important gap in the existing literature. Although previous theories have discussed the context (DIRe, SCM), motives (SST), and coping strategies (SAVI, SOC) relevant to the core mechanism of loneliness, these theories have not focused on the contents of older adults’ expectations.

## Older Adults’ Social Relationship Expectations

Social relationship expectations are shaped by personal (e.g., age-related motives and coping strategies), social, cultural, and historical contexts in a rapidly changing world (Fig. 1). Cultural expectations about intergenerational caring responsibilities (e.g., the family vs. the state as provider), conceptions of retirement (e.g., financial independence vs. filial piety), and residential settings (e.g., poverty, urban vs. rural) may affect what older adults expect from their social relationships. Although older adults tend to hold on to internalized cultural values, monumental societal changes (e.g., in population mobility, family structure, and digitalization) may be exacerbating gaps between social relationship expectations and reality (Ozawa-de Silva, 2021). Unattainable expectations could be targeted by one-on-one counseling sessions (e.g., cognitive behavioral therapy) or educational programs, such as the Friendship Enrichment Program, which helps older women clarify their expectations for friendships and has been shown to reduce loneliness (Stevens, 2001). Indeed, interventions addressing maladaptive social cognition have been shown to be the most successful type of intervention for reducing loneliness thus far, although the effect sizes of the interventions remain in the small-to-medium range (Hickin et al., 2021; Masi et al., 2011).

However, given the substantial contextual heterogeneity that characterizes loneliness in older age, interventions to reduce loneliness cannot follow a “one-size-fits-all” approach (Akhter-Khan & Au, 2020). Instead, interventions must account for the many ways in which context influences social ties (Huxhold et al., 2022). In this section, we specify six relationship expectations that older people have, including four universal expectations and two age-specific expectations that are especially salient for older adults (Fig. 2). Further, we illustrate how context shapes the contents as well as the fulfillment of older people’s relationship expectations. A comprehensive overview of social relationship expectations may fruitfully inform loneliness interventions because these interventions aim, after all, to help older adults meet their relationship expectations.

### Universal relationship expectations

#### Proximity: the availability of social contacts

To begin, older adults expect that social contacts are in proximity (e.g., ten Kate et al., 2021; Victor & Zubair, 2015). This expectation pertains to structural features, such as the quantity of social contacts an older adult has, how close by the contacts live, how available they are, and how frequently they interact with the older adult (Ashida & Heaney, 2008). Conceptually, this expectation corresponds to what has been called the “social” dimension of loneliness in previous research and scales. For instance, an item asking whether “I can call on my friends whenever I need them” appears in the social subscale of the 11-item DJG Loneliness Scale (De Jong Gierveld & Van Tilburg, 2006). Likewise, an item stating “I have nobody to talk to” is included in the UCLA Loneliness Scale (Russell et al., 1978). Being near social contacts reflects a universal, innate expectation to be embedded in a community of others, regardless of one’s life stage (Masi et al., 2011; Ozawa-de Silva, 2021; ten Bruggencate et al., 2018). Therefore, it is not surprising that most interventions for loneliness in older adults have focused on the social expectation of proximity—for example, by increasing the number of social contacts or the frequency of interactions that older adults have (O’Rourke et al., 2018).

Cultural traditions and values may influence the weight of the expectation of proximity for older adults. For instance, an expectation to have social contacts nearby is likely stronger for older adults who live in socially connected cultures that place a high value on familism than for older adults who live in individualistic cultures, in which it may be more costly to meet up with friends and family (Huxhold et al., 2022). For example, Turkish older people tend to feel more lonely when they live alone than when they co-reside with their adult children, likely because an expectation for proximity and co-residence is part of Turkish culture (ten Kate et al., 2021). Likewise, spending time with adult children and being close to them is an expectation that older South Asian people living in the United Kingdom report—an expectation that is so strong that it may even dissuade them from moving back to their home country (Victor & Zubair, 2015).

#### Support: feeling cared for and relying on others

Older adults expect to receive care and support from their social relationships (Lestari et al., 2022; Tang et al., 2020; Teerawichitchainan et al., 2015; ten Kate et al., 2021; Victor & Zubair, 2015; Zelalem et al., 2021). Items assessing one’s perception of being supported are often included in loneliness scales (e.g., “There are plenty of people I can rely on when I have problems”; De Jong Gierveld & Van Tilburg, 2006). As shown by a recent systematic review, the amount of care and support that older adults receive from others is an essential protective factor against loneliness (Dahlberg et al., 2022). Forms of support include both instrumental support (e.g., help with activities of daily living, financial assistance) and emotional support (e.g., receiving sympathy and encouragement; Lestari et al., 2022; Victor & Zubair, 2015). Moreover, both the actual amount of support that one receives and the anticipated amount of support that one feels like one could count on if needed are important aspects of this expectation—both are predictive of older adults’ sense of meaning in life (Krause, 2007). Still, a recent study showed that older adults’ loneliness was more related to unfulfilled expectations for support than to the actual amount of support that they received (ten Kate et al., 2021). In fact, this study even showed that older people with good health were lonelier than those with bad health because they (the former group) were less likely to have their expectation for support fulfilled (ten Kate et al., 2021).

The expectation of receiving support in older age from one’s children is a central aspect of the concept of filial piety. Although filial piety is present in many societies, its motivations and manifestations can have cultural inflections. In Bali, filial piety is motivated by the perception of the duty of reciprocity (Lestari et al., 2022). In South Asia, established cultural values (e.g., parental authority, affection-based relationships) contribute to filial piety (Victor & Zubair, 2015). In addition, cultures may vary in their expectations regarding who is responsible for providing care, and expectations may be gendered. Among older Bangladeshi and Pakistani residents in the United Kingdom, an expectation to be cared for by one’s spouse is stronger in men than in women (Victor & Zubair, 2015). In Vietnam, it is conventional for the oldest son of a family to provide care to older adults, whereas in Thailand, the responsibility usually lies with the oldest daughter (Teerawichitchainan et al., 2015). Given these culturally specific expectations, it is possible that older adults in Vietnam may feel lonelier when they do not co-reside with and receive care from their oldest son, whereas older adults in Thailand may feel lonelier when they do not co-reside with and receive care from their oldest daughter.

Analogously, Chinese older adults living in rural areas expect relatively more emotional support from family ties, whereas those living in urban areas expect relatively more emotional support from friendship ties (Tang et al., 2020). On the whole, cultural expectations may shape older adults’ experiences of loneliness on the basis of the correspondence between whom they receive care from versus whom they *expect* to receive care from. Modernization and urban migration, however, may be leading older adults to have lower expectations for support and lower cultural expectations for filial piety, as well as disappointment (Ren et al., 2022; Zelalem et al., 2021). Furthermore, a cross-sectional study conducted with students from several countries found that students from Asian cultures had lower intentions to care for their parents than what they believed was expected of them by their parents (Gallois et al., 1999). Aside from targeted interventions, such as functionally supporting people with impaired health, the design of age-friendly cities and policies that promote aging in place may contribute to older adults fulfilling their expectations of support and promote independence for people with limited functional ability.

#### Intimacy: feeling close, understood, and listened to

Older adults expect to have intimacy and closeness in their social relationships (Banerjee & Rao, 2022). As a foundation, older adults expect to be loved, accepted, and understood by close others (ten Bruggencate et al., 2018). A sense of trust, feelings that others are interested in one’s life (e.g., mattering), and perceptions that one can open up emotionally to others (e.g., personal validation and self-disclosure) are also important aspects of intimacy and closeness (Elliott et al., 2004; Hook et al., 2003). This expectation has often been regarded as the conceptual core of the emotional dimension of loneliness. For instance, the item “I miss having a really close friend” is included in the emotional subscale of the 11-item DJG Loneliness Scale (De Jong Gierveld & Van Tilburg, 2006), and the item “I am no longer close to anyone” is included in the UCLA Loneliness Scale (Russell et al., 1978).

An aspect of intimacy that is often overlooked among older adults is sexual intimacy. Relationship expectations concerning romantic partners—especially expectations for sexual pleasure—change with age (Banerjee & Rao, 2022). Both older men and women value sexual intimacy as an important part of their romantic relationships and life satisfaction, but age-related challenges to sexual intimacy may vary by gender (Kolodziejczak et al., 2019). For older men, impotence is a common problem that can lead to feelings of frustration within relationships (Rheaume & Mitty, 2008). Older women, in contrast, are more likely to be without a sexual partner compared with older men, partly as a result of higher rates of widowhood and longer life expectancies (Rheaume & Mitty, 2008). One qualitative study found that older adults in India understood sensuality and intimacy to mean trust, compassion, emotional support, and safety (described by one participant as “closeness . . . in body and mind”), not necessarily sexual pleasure through physical stimulation (Banerjee & Rao, 2022).

#### Fun: sharing interests and enjoyable experiences

Older adults expect to have shared interests and enjoyable experiences with others (e.g., Bantry-White et al., 2018; Heenan, 2011; van Leeuwen et al., 2019). Relevant to this, one item from the UCLA Loneliness Scale assesses whether “My interests and ideas are not shared by those around me” (Russell et al., 1978). Shared interests and enjoyable experiences may take the form of hobbies, leisure activities, or interest groups. Such activities help prevent older adults from ruminating, and they can also help the transition into retirement by giving older adults new interests, roles, and ways to spend their time (Steffens et al., 2016). Activity groups can stimulate older people’s brains (Capotosto et al., 2017), and they are often used as interventions to reduce loneliness and promote social participation among older adults (O’Rourke et al., 2018). A recent study suggested, however, that it may not be just social participation per se but rather purposeful engagement (i.e., activities that promote a sense of meaning in life) that critically reduces loneliness (Kharicha et al., 2021).

Notably, most loneliness interventions have been conducted in higher-income countries (Akhter-Khan & Au, 2020; O’Rourke et al., 2018). Older adults in low-resource settings may have comparatively fewer opportunities to engage in recreational activities or may be more limited in their participation opportunities because of financial insecurity (van Leeuwen et al., 2019). Accordingly, loneliness interventions that aim to meet the expectation to engage in enjoyable activities should account for the availability of activities in different settings. For instance, participating in discussions with other people is a universally feasible activity that may help older adults feel integrated, listened to, and stimulated. Discussion groups are often used in cognitive stimulation therapy, an intervention that aims to help people with mild cognitive impairment and dementia but that has also been shown to reduce loneliness (Capotosto et al., 2017).

### Age-specific relationship expectations

Decades of research, beginning with the foundational work of Erikson (1982), have shown that older people have especially strong concerns about *generativity*. That is, older people are especially motivated to leave a legacy, make meaningful contributions, and invest in future generations by engaging in socioemotionally meaningful activities (Tabuchi et al., 2015; Yamashita et al., 2019). Eriksonian theories of generativity have also been bolstered by evolutionary perspectives, such as Hrdy’s (2009) theory of cooperative breeding, which states that older people are evolutionarily disposed to be motivated to provide care for younger generations. Erikson (1982) hypothesized that a failure to actualize generativity may lead to negative outcomes, such as stagnation and impoverishment. Extending this hypothesis to the core mechanism of loneliness, we posit that an inability to meet generative expectations within one’s relationships, such as by caregiving or contributing, may lead to loneliness particularly among older adults. It is also possible that the respect that one receives as an older adult bears on the relations between meaningful contributions, expectations to be generative, and loneliness. A recent longitudinal study in Japan showed that older adults who felt respected by younger people were more motivated to act in generative ways compared to older adults who did not feel respected by younger people (Tabuchi et al., 2015).

However, loneliness interventions for older people have not focused on the expectation to be generative nor on the expectation to be respected and valued as an older adult (Masi et al., 2011; O’Rourke et al., 2018). These two relationship expectations are also not included in any of the most commonly used loneliness scales, such as the UCLA or DJG loneliness scales, likely because these scales were developed primarily on the basis of younger and midlife adults (De Jong Gierveld & Kamphuis, 1985; Russell et al., 1978). One reason that may explain why generativity and respect have been overlooked within loneliness research and interventions for older adults may be the misconception that older adults are primarily care receivers and costly burdens to society because of their need for long-term care, a negative portrayal that may be enhanced by ageist stereotypes (Akhter-Khan, 2021). In contrast to these stereotypes, however, older adults actually do contribute substantial amounts of economic value to society—in the form of unpaid, informal care—but their contributions are often overlooked by economic indices and thus invisible (Akhter-Khan, 2021).

Although the expectation for respect may prevail across all life stages (Elliott et al., 2004; Rosenberg & McCullough, 1981), the weight of the expectation may be age-specific, and its fulfillment may be threatened by ageism, modernization, changing cultural and family structures, functional limitations, and role loss (e.g., retirement; Ingersoll-Dayton & Saengtienchai, 1999; Victor & Zubair, 2015; Zelalem et al., 2021). Perceptions of a lack of respect may be especially pronounced for older adults in lower- and middle-income countries, in which generational attitudes regarding the expectation to respect elders have rapidly changed (Ingersoll-Dayton & Saengtienchai, 1999; Zelalem et al., 2021). This generational shift in respect for elders was described by an older Ethiopian woman in a recent study: “These days, there is no respect for [an] older person, not at all. An older person is treated like a broken utensil thrown away which is considered as useless” (Zelalem et al., 2021, p. 223).

We posit that ageism hinders the fulfillment of older adults’ relationship expectations, especially when it comes to generativity and respect. One recent study showed that aging expectations moderated the efficacy of a generativity intervention for loneliness in older adults—the intervention was more effective in older adults who had more positive expectations regarding aging (Moieni et al., 2021). The detrimental impact of ageism may also be amplified when individuals have cognitive or functional limitations. Older individuals may downplay their illnesses to protect their independence and feeling of being respected, potentially leading to neglect of one’s health (Clancy et al., 2021). In the following section, we draw on examples from different cultures and disciplines to bolster our argument that generativity and respect are two essential age-specific expectations within the core mechanism of loneliness that must be jointly considered in future loneliness research and interventions for older adults.

#### Generativity: having opportunities to contribute meaningfully

Older adults expect to be generative (i.e., to meaningfully contribute and provide care for future generations; Erikson, 1982). Generativity, as described by McAdams and de St. Aubin (1992), is motivated by both cultural demands and inner desires, and it connects the individual with the social world (Ehlman & Ligon, 2012). Although generativity can be realized through many avenues (e.g., artwork, literature, taking care of animals), human relationships are a primary avenue through which older adults actualize their expectation for generativity (Halsey & Harris, 2011). For example, generativity can be achieved through unpaid productive activities, such as caregiving, grandparenting, volunteering, and sharing life advice (Villar et al., 2021).

Longitudinal studies suggest that engaging in volunteering or grandparental care can help older adults feel less lonely (Kim et al., 2020; Tsai et al., 2013). Qualitative studies on older adults’ loneliness have also suggested that contributing meaningfully to society is an important coping strategy against loneliness (Kharicha et al., 2021). For instance, the notion of fulfillment in old age was conceptualized in terms of being able to provide care and love to children and grandchildren by an older Bangladeshi person, who hoped “to be able to continue to fulfil the parental caring role until the end of life” (Victor & Zubair, 2015, p. 113). Furthermore, taking on diverse, active, and contributing roles in society can help generate feelings of usefulness and meaning in older adults, as well as help maintain independence, autonomy, and agency (Ozawa-de Silva, 2021). This is consistent with evolutionary anthropological perspectives that propose that older adults evolved to provide care to younger generations, an intergenerational process that was instrumental for human survival (Hawkes, 2003; Hrdy, 2009). Ultimately, older adults’ expectations and motivations to provide care and contribute may benefit not only society at large but also the older adults themselves. Providing care can be a form of self-care (Akhter-Khan, 2021), a practice that results in benefits for an older adult’s own well-being, such as the reduction of loneliness.

The expectation to be generative is present across cultures (Hofer et al., 2016; Ren et al., 2022). Indeed, contributing to society is one of the five domains of functional ability that characterizes healthy aging (along with the abilities to meet basic needs, learn, grow, and make decisions, be mobile, and build and maintain relationships; World Health Organization, 2020). Yet, there may still be cultural differences in how strongly older adults expect to contribute. For instance, an anthropological study showed that feeling needed is a major expectation for social relationships and highly related to loneliness in Japan (Ozawa-de Silva & Parsons, 2020). Likewise, passing down cultural knowledge is especially important for older adults from indigenous communities across the world, in which the role of transmitting knowledge carries high status and is typically fulfilled by older people (Egeland et al., 2013; Warburton & Chambers, 2007).

However, old age and retirement are viewed in Chinese culture as a phase of life in which older people may take a more passive role and be cared for by their children. Having worked hard their whole lives, and having successfully raised children who are filial and financially secure enough to support their parents, Chinese older adults are encouraged to rest instead of continuing to actively contribute (Luo & Chui, 2016). Thus, cultural conceptions of aging and retirement may shape how strongly the expectation for generativity may be felt. One open challenge is how to design opportunities to contribute for older people with impaired functional ability. Initiatives such as the Japanese “Restaurant of Mistaken Orders,” which employs people with dementia, may help promote positive aging attitudes and give older adults the opportunity to fulfill their expectation to be generative.

#### Respect: feeling valued and actively included

Finally, older adults expect to be respected and valued by others (ten Kate et al., 2021; Victor & Zubair, 2015; Zelalem et al., 2021). On the micro level, referring to social relationships with friends and family members, older adults expect to be valued for their contributions (e.g., the care that they provide to family members) and included in activities and decision-making (Gallois et al., 1999; Ingersoll-Dayton & Saengtienchai, 1999; ten Kate et al., 2021; Victor & Zubair, 2015; Warburton & Chambers, 2007). On the meso level, referring to interactions with others in the neighborhood, public spaces (e.g., markets or healthcare settings), and broader society, older adults expect to be treated as elders who deserve respect, politeness, and dignity (Clancy et al., 2021; Van Der Geest, 2004; Warburton & Chambers, 2007).

On both the micro and meso levels, the amount of respect older adults receive may correspond to their social status (e.g., adults with lower social status may receive less respect). One study showed that older adults who perceived their social status to be low experienced more loneliness than those who perceived their social status to be high (Yu et al., 2021). Moreover, not being respected may lead to the social exclusion or abuse of older adults, both of which are strongly linked to loneliness (Burholt et al., 2020). On the macro level, older adults expect to be represented and included in political and societal decision-making (e.g., Hodgkin, 2012). Unfortunately, although older people contribute immensely to society by volunteering and providing informal unpaid care, their contributions are often overlooked by economic indices (e.g., gross domestic product) and are thus not visibly valued or centrally considered in public policy making (Akhter-Khan, 2021; Warburton & McLaughlin, 2005).

Several studies show that the cultural expectation to be respected as an older adult is strong in many cultures, such that even younger adults are aware of this expectation. In one study, for example, students from several Asian cultures (Japan, Korea, Hong Kong, and the Philippines) believed that older adults would expect continued contact and respect from them (Gallois et al., 1999). Another cross-sectional study showed that Korean students reported a wider variety of culturally specific forms of respect for older adults compared with North American students (Sung, 2004). These culturally specific forms of respect included care respect (e.g., caring by living together with older people), consulting respect (e.g., consulting older adults over personal and family matters), linguistic respect (e.g., using honorifics to address older adults), and victual respect (e.g., serving drinks and foods of older people’s choice), among others (Sung, 2004).

Although respect may take many forms, older adults may have an age-specific expectation for a type of respect that is tied to being recognized and valued for one’s generative contributions (Tabuchi et al., 2015). For instance, in Nordic countries, in which a high value is placed on treating people with dignity in healthcare settings, older adults may still feel that they are missing a lack of recognition and confirmation of their worth and contributions to society (Clancy et al., 2021). Likewise, older adults in Ghana are generally treated with politeness, but they may still feel lonely if they are not respected for sharing wisdom and advice (Van Der Geest, 2004). In a qualitative study from Ethiopia, older adults reported being treated as useless assets, which gave them a feeling of not being valued (Zelalem et al., 2021).

## Responding to Loneliness

According to the SRE framework, whether a person feels lonely or not depends on whether the six relationship expectations are met. Importantly, loneliness is not necessarily a permanent condition from which one cannot recover. Whereas some individuals do experience persistent or *chronic loneliness*, other individuals experience *transient loneliness*, from which they recover (as indicated by the blue pathway in Fig. 1). This conceptualization of transient loneliness is in line with the Evolutionary Theory of Loneliness (ETL; Cacioppo et al., 2006, 2014). The ETL posits that loneliness is not necessarily a negative chronic state; instead, it can be a transient state that serves the adaptive function of motivating behavior change (e.g., by motivating the repair of one’s social connections that are needed for sustaining health and well-being; Cacioppo et al., 2014). Supporting the distinction between transient and persistent loneliness, studies have shown that these two types of loneliness have different effects on older adults’ health (Akhter-Khan et al., 2021; Shiovitz-Ezra & Ayalon, 2010; Zhong et al., 2016).

Loneliness may be transient when one successfully uses various coping strategies, such as those described by SOC. Importantly, the specific forms that these coping strategies take are always context-dependent, and the conceptualization and stigmatization of loneliness in a given culture or religion may naturally affect how older adults cope. For instance, qualitative studies have shown that older people’s use of coping strategies to overcome loneliness may vary depending on cultural context (Rokach et al., 2004; Rokach & Neto, 2005), as well as functional ability (Schoenmakers et al., 2012).

As seen in Figure 1, when people are in a context that does not allow them to successfully apply coping strategies, they may experience persistent loneliness. Personal cognitive and behavioral processes that lead to chronic loneliness have been previously described elsewhere (Käll et al., 2020). However, there are also important contextual factors that may lead to chronic loneliness. Impaired health and financial insecurity are two common predictors of loneliness identified by recent systematic reviews, which mainly included participants from high-income countries (Cohen-Mansfield et al., 2016; Dahlberg et al., 2022). It is plausible that persistent loneliness is an even bigger problem in low-resource settings in which older people depend on their job and income for ensuring that their basic needs are met (Gonyea et al., 2018). In Indonesia, for example, participation rates in economic activities for older adults is 66%, approximately twice as high as in European countries such as England or France (Ko & Yeung, 2019). Impaired health is one of the biggest obstacles to engaging in economic activities in rural areas and may especially detrimental when there is no governmental support system to lend assistance (Utomo et al., 2019). Accordingly, people with age-related functional decline who live in low-resource settings without financial support (from governmental programs or family members) may not be able to effectively use coping strategies such as selecting new goals to reduce loneliness because their opportunities may be limited by their structural contexts (e.g., their dependence on economic engagement) (Akhter-Khan et al., in press). Therefore, poverty, or a lack of financial support from family members or the government, may exacerbate the experiences of both shame and loneliness (Ma et al., 2019). Specific attention should be given to marginalized older populations in low-resource settings, who are often overlooked in studies on loneliness (e.g., Dahlberg, 2021).

## Future Directions for Research and Interventions

The SRE framework may inform further research, clinical practice, and interventions that aim at reducing older adults’ loneliness. It is especially suited for person-centered approaches, such as the precision-health approach, which aims to deliver the right solution to the right person at the right time (Akhter-Khan & Au, 2020). The specification of the six different relationship expectations makes it possible to assess, for a given person, which expectation they may need the most help with at a given time, which in turn enables tailoring existing interventions to meet specific expectations. An important question for future studies is how the six expectations in the SRE framework may interact across the life span, given that efforts to meet the different expectations may compete for an individual’s time, attention, and resources. In addition to qualitative research, creating an assessment tool to measure the six expectations may help shed light on how different dimensions of the SRE framework interact and how they are related to personal characteristics (e.g., age, gender), cultural norms, and aging expectations. Assessments based on the SRE framework could also help test the pathways pertaining to the core mechanism of loneliness (Fig. 1) across different life stages. Although the SRE framework relies on the psychological (i.e., cognitively oriented) definition of loneliness, it may also be suitable for integrating more diverse, anthropological conceptualizations of loneliness (Ozawa-de Silva & Parsons, 2020).

As highlighted by the UN Decade of Healthy Aging 2021–2030 baseline report, programs and interventions need to be informed by evidence and aligned with older people’s expectations (World Health Organization, 2020). Generativity and respect are two expectations in this framework that have not been adequately addressed in previous loneliness interventions for older people. To successfully implement person-centered approaches, reduce loneliness, and promote healthy aging around the world, efforts must be made to understand how cultural demands shape conceptions of and avenues for fulfilling generativity in underrepresented regions, as well as how older adults can be valued for their contributions (Villar et al., 2021). This entails a need for more research in different cultural contexts, especially lower- and middle-income countries, in which the prevalence of loneliness is high, but research and programs aimed at reducing loneliness are limited (Gao et al., 2021; Surkalim et al., 2022). Valuing older adults’ contributions may be an important avenue for loneliness interventions, one that may efficiently address both older adults’ expectation for generativity and their expectation for respect.

Promoting generativity and opportunities to fulfill generative expectations may lead to benefits for older adults as well as society at large and may even help transform population aging into a powerful force for addressing pressing challenges (Akhter-Khan, 2021). Engaging older adults in climate change action and volunteering may be one way to reduce older adults’ loneliness, but there have been few investigations in this direction, which means that a substantial research gap remains (Pillemer et al., 2021). Participatory action research (PAR) is a promising approach for achieving many important aims in tandem: developing inclusive interventions to reduce older adults’ loneliness, respecting and valuing older adults’ contributions visibly, and involving older adults in community decision-making. Indeed, an integrative review of loneliness interventions showed that community development approaches, in which older adults were engaged in decision-making processes during an intervention, were more effective at reducing loneliness than typical top-down interventions (Gardiner et al., 2018). PAR methods may involve storytelling, oral history projects, sharing life experiences, and photovoice (Ehlman & Ligon, 2012; Moieni et al., 2021). Another underexplored topic is how communication technologies could help older adults meet their expectations, promote intergenerational contact, and provide opportunities for older adults to be seen, heard, and valued in their communities and beyond.

In addition to informing interventions, the SRE framework could also inform research into pressing questions on loneliness: why some people experience chronic loneliness (whereas others are able to overcome this feeling), how chronic loneliness can be prevented in diverse contexts, and how coping strategies can be promoted among people who feel chronically lonely, especially in populations in which loneliness is stigmatized.

## Conclusion

Reducing loneliness by addressing older adults’ social relationship expectations represents one pathway towards promoting functional ability and, ultimately, healthy aging. The SRE framework highlights the need to create opportunities for older people to contribute and fulfill their expectations for generativity, as well as opportunities to recognize, value, and respect the contributions of older people. Participatory approaches may be a promising avenue for future research and interventions for loneliness in older adults and may also help fulfill older adults’ expectations to be seen, heard, and valued. Looking ahead, reducing loneliness will require interdisciplinary collaborations, especially those that can draw upon the culturally specific wisdom and input of older people. The best place to find solutions for meeting older people’s relationship expectations may be in the thoughtful advice and participation of older people themselves.

## Transparency

*Action Editor:* Tina M. Lowrey

*Editor:* Klaus Fiedler
